# Toxicities of docetaxel: original drug versus generics—a comparative study about 81 cases

**DOI:** 10.1186/s40064-016-2351-x

**Published:** 2016-06-16

**Authors:** Choukri Elm’hadi, Rachid Tanz, Mohamed Reda Khmamouche, Mehdi Toreis, Tarik Mahfoud, Khaoula Alaoui Slimani, Hassan Errihani, Mohammed Ichou

**Affiliations:** Medical Oncology Department, Mohammed V Military Teaching Hospital of Rabat, Rabat, Morocco; Medical Oncology Department, National Institute of Oncology Sidi Mohamed Ben Abdellah, Rabat, Morocco; School of Medicine and Pharmacy, University Mohamed V, Souissi, Rabat, Morocco

**Keywords:** Docetaxel, Generic, Toxicity, Cancer

## Abstract

**Introduction:**

Docetaxel is a chemotherapy drug widely prescribed in oncology that recognizes a variety of manufactured generics whose toxicity is increasingly reported. The aim of this study was to compare the toxicities between the original and a generics docetaxel in a Moroccan center.

**Methods:**

In a cross sectional study, we enrolled patients treated with docetaxel from the oncology department of the military hospital of Rabat over a period of 2 years (2013–2014). We compared the prevalence of hypersensitivity reactions, febrile neutropenia, peripheral neuropathy, gastrointestinal, cutaneous, and hematologic toxicities, between four different presentations of docetaxel including the original drug. Only grade II or worse adverse events related to chemotherapy were considered. Treatments discontinuations due to toxicity were also compared. Unusual skin toxicities were included.

**Results:**

81 patients were eligible for analysis [43/generics arm vs. 38/original drug arm. Hematological toxicity was significantly more frequent in the generic arm than in the original drug (32.6 vs. 13.2 %; *p* = 0.04)]. Also, a signifying higher rate of treatment discontinuation was observed in the generic arm (39.5 vs. 7.9 %, *p* = 0.001). The use of specific generic increase numerically the skin toxicities (17.6 vs. 0 %, *p* = 0.026).

**Conclusion:**

Our data suggest that generics of docetaxel are associated with an increase of hematological and cutaneous toxicities, an increase of treatment discontinuation rate and emphasize the need of a regulation of generics’ manufacture.

## Background

Docetaxel is a second-generation taxane that produces a cytotoxic effect by inhibiting depolymerization of microtubules which, in turn, inhibits cell replication. This drug has also been shown to inhibit angiogenesis, induce signaling aberrations, and induce mitotic catastrophe or apoptosis (Herbst and Khuri 2003). Docetaxel was developed by sanofi-aventis and is registered and marketed in the form of an injectable solution under the brand name Taxotere*. It is highly effective as monotherapy and combination therapy across a variety of tumor types including breast cancer in its various stages, non-small cell lung cancer, androgen-independent metastatic prostate cancer, head and neck cancers, gastric cancer and other indications except approval (Sanofi-Aventis U.S. LLC 2011). The most common severe adverse reactions (grade III–IV) related to docetaxel (100 mg/m^2^) as a single agent are asthenia (12.8 %), cutaneous reactions (4.8 %), fluid retention (6.9 %), gastrointestinal reactions (2.7–4.7 %), hypersensitivity reactions (4.2 %), neurosensory reactions (4.3 %), neuromotor reactions (3.6 %), stomatitis (5.5 %), anemia (8.8 %), febrile neutropenia (11.0 %), infections (6.1 %), leucopenia (31.6 %), and neutropenia (75.4 %) (Sanofi-Aventis U.S. LLC 2011).

Due to its multiple indications, docetaxel recognizes a variety of generic used to modulate the economic cost (Generic Pharmaceutical Association 2009). Generic drugs are a huge and complex part of the health-care market. Cancer drugs are no exception. In a total global oncology drugs market approaching $100 billion, revenues from generics are growing at twice the rate of the market as a whole, the vast majority of all drug prescriptions are already for generics more than 80 % in the United States, for example Generic Pharmaceutical Association (2009).

To obtain Moroccan approval (Décret n° 2-12-198 du 21 rejeb 1433, 2012), a generic drug must prove pharmaceutical properties equivalent to those of a reference drug in terms of efficacy and safety. This bioequivalence recommended the same amount of active ingredient, in the same dosage and using the identic route of administration, and comply with accepted standards of quality, purity in accordance with international guidelines (Food and Drug Administration 2012). However; phase IV post-marketing studies were not required to have approbation.

Differences in generic drug formulations have been noted, and probably have an impact on drugs with a narrow therapeutic index as a docetaxel. Toxicity of generic docetaxel is increasingly reported in the literature and is a subject of heated debate and controversy. Impurities, excipients, and the amount of active agent itself can all have an impact on the efficacy and have been implicated in the occurrence of these side-effects (Vial et al. 2008; Garrido-Siles et al. 2015).

Also, following the introduction of these new formulations of docetaxel, our oncology department recorded a higher prevalence of differents toxicities reported with atypical symptomatology and pronounced grades causing an imbalance of cost/benefit. The aim of this study was to assess the frequency of grade II, III and IV adverse events and discontinuation treatment due to toxicity between original docetaxel and a generics formulation in a Moroccan department of medical oncology.

## Methods

### Study design

This is a retrospective study over a period of 2 years (from 1 January 2013 to 31 December 2014) including patients who were treated with docetaxel at the medical oncology department of the military hospital in Rabat in Morocco. Patients were assigned into 4 groups. Group 1 receiving original drug, group 2 receiving the first generic (French origin), group 3 receiving the second generic (Australian-American origin) and group 4 receiving the third generic (Indian origin). We compare the incidence of hypersensitivity reactions, gastrointestinal, cutaneous, hematological toxicities, febrile neutropenia and peripheral sensory neuropathy to four different presentations of docetaxel including the original drug. Only grade II or worse adverse events related to chemotherapy should be considered according to the classification NCI CTCAE 4.0 (national cancer institute common terminology criteria for adverse events) (U.S. Department of Health and Human Services 2009). Treatment discontinuation due to toxicity is also compared. Unusual skin toxicities with are included. Toxicities grade III or worse were declared to the national pharmacovigilance Centre.

Hypersensitivity reactions were categorized according to CTCAE 4.0 criteria, considering as an event any grade II or more allergic reaction, requiring immediate discontinuation of the infusion of docetaxel and appropriate treatment to counteract the reaction.

Hematologic toxicities include anemia with hemoglobin less than 10.0 g/dL, neutropenia with neutrophils less than 1500 cells/mm^3^, leukopenia with white blood cells less than 4000 cells/mm^3^, and thrombocytopenia with platelets less than 100,000 cells/mm^3^.

Febrile neutropenia was defined by neutrophils less than 1000/mm^3^ with a single temperature of >38.3 °C or a sustained temperature of ≥38 °C for more than 1 h.

Patients treated with TPF Protocol have benefited from a pegfilgrastim primary prophylaxis from days 5 to 11 whatever the drug used.

Gastrointestinal toxicities include nausea, vomiting more than two episodes in 24 h, and diarrhea increase of four stools per day.

Peripheral sensory neuropathy was defined by symptoms limiting instrumental or self care of activity daily living.

Skin toxicity include hand and foot syndrome limiting instrumental or self care of activity daily living.

Unusual skin toxicities are defined as being toxicities related to the use of chemotherapy that are not described in the monograph of the original drug like localized erythema of the extremities (palm of the hands and soles of the feet) with edema, followed by desquamation.

Patients routinely receive premedication with oral corticosteroids started 1 day before chemotherapy and maintained 3 days after (prednisolone 1 mg per kg), with intravenous infusion on the day of chemotherapy (methylprednisolone 120 mg bolus 30 min before docetaxel).

Excluded patients are those that have less than grade II toxicity. Other reasons for exclusion included the absence of premedication and patients in which the molecule used was not mentioned.

### Data collection

Informations on demographic characteristics and medical history were collected using the clinical files in all cases with determination of the age, sex, cancer diagnosis, chemotherapy protocol, number of courses, drug involved and type of toxicity. Unusual skin toxicities are retained after a dermatological consultation confirming the responsibility of drugs used and eliminating other possible causes.

#### Statistical analysis

All analyses were performed with SPSS 18 software. The number of patients and the corresponding percentages were given for categorical variables, mean ± standard deviation were reported to describe the normally distributed continuous variables, and medians with interquartile ranges were reported for continuous variables with skewed distributions. The Kolmogorov–Smirnov test was performed on all measures to assess data normality. Chi square or Fisher’s exact test were used to compare the categorical variables as appropriate. Means were compared using the Student’s *t*-test and medians were compared using the Mann–whitney test. Univariate logistic regression analysis was conducted to compare several toxicities between the original drug and generics. A *p* value <0.05 was considered statistically significant.

## Results

During the period of this study, 282 courses of chemotherapy docetaxel were administered to 81 patients. 38 have received the original drug and 43 patients received one of three presentations of generics, 15 patients received the first generic, 17 patients received the second generic), and 11 patients received the third generic. The mean age of the patients was 48.3 ± 9.2 years with a female predominance (79 %). Baseline characteristics of patients are summarized in Table 1. The most frequent diagnosis was breast cancer (79 %), and the following chemotherapy regimens were most commonly used: 5FU-epirubicin-cyclophosphamide (three cycles) followed by docetaxel (three cycles) (3FEC100-3taxotere scheme) (54.3 %), docetaxel–trastuzumab combination (12.4 %), and docetaxel monotherapy (28.4 %).

**Table 1:** Patients’ baseline characteristics

	Original drug (n = 38)	Generic 1 (n = 15)	Generic 2 (n = 17)	Generic 3 (n = 11)	Total (n = 81)
Age (ans)^a^	46.2 ± 8.2	48.5 ± 8.7	53 ± 10.7	48.1 ± 9.2	48.3 ± 9.2
Sex					
Male	6 (15.8 %)	2 (13.3 %)	7 (41.2 %)	2 (18.2 %)	17 (21 %)
Female	32 (84.2 %)	13 (86.7 %)	10 (58.8 %)	9 (81.8 %)	64 (79 %)
Cancer diagnosis					
Early breast	26 (68.4 %)	9 (60 %)	9 (52.9 %)	8 (72.7 %)	52 (64.2 %)
Metastatic breast	6 (15.8 %)	4 (26.7 %)	1 (5.9 %)	1 (9.1 %)	12 (14.8 %)
Gastric	2 (5.3 %)	0	2 (11.8 %)	0	4 (4.9 %)
Head and neck	1 (2.6 %)	2 (13.3 %)	1 (5.9 %)	0	4 (4.9 %)
Lung	1 (2.6 %)	0	2 (11.8 %)	0	3 (3.7 %)
Prostate	2 (5.3 %)	0	2 (11.8 %)	1 (9.1 %)	5 (6.2 %)
Sarcoma	0 (0 %)	0	0	1 (9.1 %)	1 (1.2 %)
Chemotherapy protocol					
T	9 (23.7 %)	4 (26.7 %)	7 (41.2 %)	3 (27.3 %)	23 (28.4 %)
FEC-T	22 (57.9 %)	7 (46.7 %)	8 (47.1 %)	7 (63.6 %)	44 (54.3 %)
FEC-TH	4 (10.5 %)	2 (13.3 %)	1 (5.9 %)	1 (9.1 %)	8 (9.9 %)
TPF	1 (2.6 %)	2 (13.3 %)	1 (5.9 %)	0	4 (4.9 %)
TH	2 (5.3 %)	0	0	0	2 (2.5 %)
Number of chemotherapy courses^b^	3 [3, 5]	3 [2, 3]	3 [3, 4.5]	3 [2, 3]	3 [3, 4]

*T* taxotere, *FEC* 5-fluorouracil + epirubicin + cyclophosphamide, *TH* docetaxel + trastuzumab, *TPF* docetaxel + cisplatin + 5-fluorouracil

^a^Mean ± standard deviation

^b^Median [interquartile range]

Table 2 shows the comparative analysis of toxicities observed in both arms.

**Table 2:** Comparative analysis of the toxicity of docetaxel: original drug versus generics

Toxicities	Original drug (n = 38)	Generics (n = 43)	*p*
Hypersensitivity	1 (2.6 %)	0 (0 %)	0.46
Peripheral sensory neuropathy	2 (5.3 %)	7 (16.3 %)	0.16
Hematological toxicities	5 (13.2 %)	14 (32.6 %)	*0*.*04*
Febrile neutropenia	1 (2.6 %)	4 (9.3 %)	0.36
Gastrointestinal toxicity	2 (5.3 %)	6 (14 %)	0.27
Skin toxicity	0 (0 %)	5 (11.6 %)	0.05
Extra nail hypermelanosis	0 (0 %)	1 (2.3 %)	1
Hand foot syndrome grade III	0 (0 %)	3 (7 %)	0.24
Ichthyosis-like eruption	0 (0 %)	1 (2.3 %)	1
Treatment discontinuation	3 (7.9 %)	17 (39.5 %)	*0*.*001*

Italic values indicate statistical significants

Toxicities of each formulation were evaluated in comparison with the original drug in the Tables 3, 4 and 5.

**Table 3:** Comparative analysis of toxicity: original drug versus generic no. 1

	Original drug (n = 38)	Generic no. 1 (n = 15)	*p*
Hypersensitivity	1 (2.6 %)	0 (0 %)	1
Peripheral sensory neuropathy	2 (5.3 %)	2 (13.3 %)	0.56
Hematological toxicities	5 (13.2 %)	3 (20 %)	0.67
Febrile neutropenia	1 (2.6 %)	1 (6.7 %)	0.49
Gastrointestinal toxicity	2 (5.3 %)	2 (13.3 %)	0.56
Skin toxicity (hand foot syndrome grade III)	0 (0 %)	1 (6.7 %)	0.28
Treatment discontinuation	3 (7.9 %)	4 (26.7 %)	0.09

**Table 4:** Comparative analysis of toxicity: original drug versus generic no. 2

	Original drug (n = 38)	Generic no. 2 (n = 17)	*p*
Hypersensitivity	1 (2.6 %)	0 (0 %)	1
Peripheral sensory neuropathy	2 (5.3 %)	2 (11.8 %)	0.58
Hematological toxicities	5 (13.2 %)	4 (23.5 %)	0.43
Febrile neutropenia	1 (2.6 %)	1 (5.9 %)	0.52
Gastrointestinal toxicity	2 (5.3 %)	3 (17.6 %)	0.16
Skin toxicity	0 (0 %)	3 (17.6 %)	*0*.*026*
Hand foot syndrome grade III	0 (0 %)	2 (11.8 %)	0.09
Ichthyosis-like eruption	0 (0 %)	1 (5.9 %)	0.3
Treatment discontinuation	3 (7.9 %)	5 (29.4 %)	0.09

Italic value indicates statistical significants

**Table 5:** Comparative analysis of toxicity: original drug versus generic no. 3

	Original drug (n = 38)	Generic no. 3 (n = 11)	*p*
Hypersensitivity	1 (2.6 %)	0 (0 %)	1
Peripheral sensory neuropathy	2 (5.3 %)	3 (27.3 %)	0.06
Hematological toxicities	5 (13.2 %)	7 (63.6 %)	*0*.*002*
Febrile neutropenia	1 (2.6 %)	2 (18.2 %)	0.12
Gastrointestinal toxicity	2 (5.3 %)	1 (9.1 %)	0.54
Skin toxicity (extra nail hypermelanosis)	0 (0 %)	1 (9.1 %)	0.22
Treatment discontinuation	3 (7.9 %)	8 (72.7 %)	*<0*.*001*

Italic values indicate statistical significants

The hematologic toxicity was more frequent in the generic arm than in the original drug (32.6 vs. 13.2 %, *p* = 0.04). Furthermore, higher rate of treatment discontinuation was observed in the generic group with a rate of 39.5 % versus 7.9 % in the original drug (*p* = 0.001) (Table 2).

In univariate analysis, hematologic toxicity (OR 3.18 95 % IC [1.02; 9.92]; *p* = 0.046) and treatment discontinuation (OR 7.62 95 % IC [2.02; 28.78]; *p* = 0.003) were significantly associated with the generics’ use (Table 6).

**Table 6:** Simple logistic regression examining the toxicities associated with generics

	Univariate analysis		
	OR	IC 95 %	*p*
Peripheral sensory neuropathy	3.5	0.68, 18	0.13
Hematological toxicities	3.18	1.02, 9.92	*0*.*046*
Febrile neutropenia	3.8	0.4, 35.54	0.24
Gastrointestinal toxicity	2.9	0.55, 15.42	0.2
Treatment discontinuation	7.62	2.02, 28.78	*0*.*003*

Italic values indicate statistical significants

In subgroups analysis, only the third generic was significantly associated with an increase of hematological toxicities (63.6 vs. 13.2 %; *p* = 0.002) and treatment discontinuation (72.7 vs. 7.9 %; *p* < 0.001) (Table 5).

No leukopenia or neutropenia has been reported in patients who received pegfilgrastim primary prophylaxis. The use of this prophylaxis did not influence the results obtained [2.67 % in the original group versus 7 % in the generic group, *p* = 0.61(Table 1)].

A grade II immediate hypersensitivity reaction occurred in one patient during infusion of the first course of docetaxel (original drug) requiring discontinuation treatment (Table 2). The symptoms reported were skin rash, flushing, tachycardia, dizziness and hypotension. Outcome was favorable after fluid replacement, intravenous paracetamol, corticosteroid and hydroxyzine. Retreatment with docetaxel was well tolerated after decreasing the infusion rate.

**Fig. 1:**
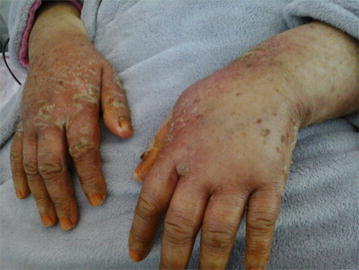
Hand foot syndrome grade III in a patient prescribed generic drug formulation 3

**Fig. 2:**
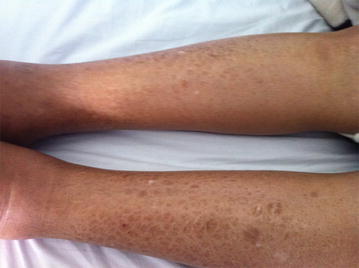
Ichthyosis-like eruption of the lower limbs in a patient prescribed generic drug formulation 2

No skin toxicity was observed in patients treated by original drug (Table 2). However, severe hand foot syndrome grade III was observed in three patients of generic group requiring discontinuation treatment (Fig. 1). Moreover, two patients of the same group have developed unusual skin toxicities: extra nail hypermelanosis sitting in the abdominal wall evolving favorably after corticosteroids and antihistamines medication in a patient, and ichthyosis-like eruption of the lower limbs in the other (Fig. 2). Cutaneous toxicities were significantly more frequent in patients treated by the second generic compared to the original drug (17.6 vs. 0 %; *p* = 0.026) (Table 4).

## Discussion

Our study shows an increase of hematological toxicities, skin toxicities, and increase of treatment discontinuation rate especially with two of three generics used. There are few studies in the literature that have investigated the toxicity of the docetaxel’s generics. Siles-Garrido et al. report a significant increase in the incidence of acute infusion reactions and unusual irritative skin toxicity in patients treated with three formulations of docetaxel (Garrido-Siles et al. 2015). Skin lesions observed in this study appear early, with a purplish red inflammatory character, painful and perivenous topography adjacent to the drug administration site. These lesions were similar to extravasation with a less favorable response to corticosteroids. This toxicity was seen in 9 % of patients, including 1.5 % who required dose adjustment. In our series, no acute infusion reactions have been reported with generics drugs. However, skin toxicity was seen in 11.5 % of cases with predominance of hand-foot syndrome grade III. Also, two unusual skin toxicities were observed: extra nail hypermelanosis and ichthyosis-like eruption. The incidence of grade III/IV skin toxicities described for original drug varies from 0.8 to 5.9 % depending on the dose used (Chew and Chuen 2009). Elementary lesions are erythematous maculopapular rash on the extremities, face, chest and arms.

A recent retrospective study carried out in Canada compared just one generic (second generic in our study) with the original in 364 patients with breast cancer found no difference between groups for occurrence of intestinal perforations, thrombotic events, hand and foot syndromes, and docetaxel-related deaths. The number of febrile neutropenic events was similar in both groups (original, 17.6 %, vs. generic, 18.1 %; *p* = 0.89 and *p* = 0.71 after adjustment for context, use of G-CSF (granulocyte colony-stimulating factor), docetaxel dose, and age, using a Poisson model. However, the proportion of grade IV febrile neutropenia was higher in the generic group (original, 56.3 %, vs. generic, 78.8 %; *p* = 0.05 and *p* = 0.06 after adjustment for context, use of G-CSF, docetaxel dose, and age, using a Poisson model (Poirier et al. 2014).

Two factors could contribute to the explanation of these equivalent results: firstly, the present study may suffer from some certain weaknesses that make patients in the generic arm received more growth factors, and then this drug is likely to be of high quality, given it is on the Canadian market, which is said to have one of the world’s best bio-equivalence inspection regimes. However, patients who received this generic needed prolonged hospitalization and increased use of granulocyte growth factors, suggesting the loss of the economic benefit to use generic drugs (Poirier et al. 2014).

Several theories have attempted to explain the toxicities with generic docetaxel based on pharmacological characteristics. The mechanisms involved were underdosing docetaxel, high level of impurities and excipients used.

Levels of docetaxel were evaluated by Vial et al. on generic formulations purchased in 14 countries (Vial et al. 2008). The results were surprising 21 generics contained less than 90 % docetaxel, 11 of which had less than 80 % of what would be expected. Only 10 were in the acceptable range of 90–110 %. In referring to this study, the first and third generics used in our study were away the acceptable range. The second generic was not available at the time of the study.

Levels of impurities were also obtained in same study using reverse-phase liquid chromatography with ultraviolet detection setting a conservative limit of 3 % (the reference was 1.6 %). They found that 23 of the generics had impurities levels of more than 3 % and one of the generics, from India, had a 20 % level of impurities. Importantly, 33 of the impurities were not detected at all in the reference which that we do not know the physiological role and clinical consequences. In our study, the first and the third generic employed have high levels impurities (which one had a more than 6 %).

The role of excipients is also reported, docetaxel is a hydrophobic drug that requires solvents to improve its solubility and allow its constitution. The solubiliser used is polysorbate 80 (Tween 80), a non-ionic surfactant vehicle whose main constituent is polyoxyethylene-20-sorbitanmonooleate which is structurally equivalent to polyethylene glycols (PEG). Nevertheles, polysorbate 80 is a pharmacologically and biologically active excipient associated with side effects such as hypersensitivity reactions, peripheral neuropathy (Tije et al. 2003), vascular toxicity and fluid retention (Drori et al. 1995; Mark et al. 2001).

The other excipient mostly described in the literature is ethanol, a substance whose presence must be indicated on the drug that contains it. Cases of alcohol poisoning have been reported in patients receiving high doses of ethanol or in pediatric population, who are more susceptible to the effects of this substance (Zuccotti and Fabiano 2011). The food and drug administration (FDA) is warning that the chemotherapy drug docetaxel contain ethanol, also known as alcohol, which may cause patients to experience intoxication or feel drunk during and after treatment (U.S. Food and Drug Administration 2014). The warning was issued after a review of the FDA database and the data in the medical literature about adverse effects, which identified three cases of alcohol intoxication associated with various presentations of docetaxel (Mirza and Mithal 2011). Two incidents occurred during the infusion; one patient developed symptoms within 24 h.

Eight formulations of docetaxel have been identified by the FDA including generic and brand-name products, from several manufacturers. The alcohol content in each 200-mg dose ranges from a low of 2 g in original drug to a high of 6.4 grams in docetaxel injections manufactured by Pfizer (U.S. Food and Drug Administration 2014).

In Australia, Pfizer’s docetaxel application was withdrawn before a final decision by the country’s regulator. In contrast, the United Kingdom, and the United States have both authorized this generic.

The alcohol content in a dose of docetaxel may affect the central nervous system and should be taken into account for patients in whom alcohol intake should be avoided or minimized, including patients with hepatic impairment. Docetaxel formulation with the lowest possible alcohol content should be considered for patients who experience adverse reactions (U.S. Food and Drug Administration 2014). Patient may also have religious objections.

Clinically, the Garrido-Siles study (2015) suggested that some skin toxicities of docetaxel may be caused by the excipients used in the different generics. There seems to be a relationship between the lower content of polysorbate 80 and the lower incidence of severe hypersensitivity reactions. However, administration of presentations with higher ethanol content is associated with increased incidence of skin toxicity, occasionally severe, near the injection site of the drug.

There was also a significant increase in the incidence of skin toxicity following the administration of the second generic (17.6 vs. 0 %; *p* = 0.026). New cutaneous toxicities with generic docetaxel were observed, but relationship with content of ethanol or polysorbate 80 was not assessed.

While the results of the study are statistically significant, it is premature to make definitive conclusions on the quality of generic docetaxel from various sources. Nevertheless, the data are supportive of other studies suggesting that the formulation of generic docetaxel is important to consider, as some appear to be increasing host toxicity in a vulnerable patient population.

The present study has some methodological weaknesses. They are the small sample size and the retrospective nature of the study, including heterogeneous baseline demographic and disease characteristics. These include age, sex, stage, disease site and comorbidities which could have induced bias in the case selection or events reporting. Moreover, different chemotherapy regimens and generics were used, with unavailability of information on levels of impurities, levels of docetaxel and the excipients used. These factors should be considered in future studies.

## Conclusion

Our data suggest that generics of docetaxel are associated with an increase of hematological toxicities, cutaneous toxicities, and an increase of treatment discontinuation rate for which the exact mechanism is unknown. Serious adverse events, requires longer hospitalization and the economic benefit of using a generic formulation may be lost. So, therapeutic changes could impact the overall survival of patients. International harmonization of regulations in the manufacture of generics should be encouraged.
